# Monocyte-derived macrophages contain persistent latent HIV reservoirs

**DOI:** 10.1038/s41564-023-01349-3

**Published:** 2023-03-27

**Authors:** Rebecca T. Veenhuis, Celina M. Abreu, Pedro A. G. Costa, Edna A. Ferreira, Janaysha Ratliff, Lily Pohlenz, Erin N. Shirk, Leah H. Rubin, Joel N. Blankson, Lucio Gama, Janice E. Clements

**Affiliations:** 1 Department of Molecular and Comparative Pathobiology, Johns Hopkins University School of Medicine, Baltimore, MD USA; 2 Department of Neurology, Johns Hopkins University School of Medicine, Baltimore, MD USA; 3 Department of Epidemiology, Johns Hopkins University School of Medicine, Baltimore, MD USA; 4 Department of Psychiatry and Behavioral Sciences, Johns Hopkins University School of Medicine, Baltimore, MD USA; 5 Department of Medicine, Johns Hopkins University School of Medicine, Baltimore, MD USA; 6 Vaccine Research Center, National Institute of Allergy and Infectious Diseases (NIAID), National Institutes of Health (NIH), Bethesda, MD USA; 7 Department of Pathology, Johns Hopkins University School of Medicine, Baltimore, MD USA

**Keywords:** HIV infections, Molecular biology

## Abstract

The development of persistent cellular reservoirs of latent human immunodeficiency virus (HIV) is a critical obstacle to viral eradication since viral rebound takes place once anti-retroviral therapy (ART) is interrupted. Previous studies show that HIV persists in myeloid cells (monocytes and macrophages) in blood and tissues in virologically suppressed people with HIV (vsPWH). However, how myeloid cells contribute to the size of the HIV reservoir and what impact they have on rebound after treatment interruption remain unclear. Here we report the development of a human monocyte-derived macrophage quantitative viral outgrowth assay (MDM-QVOA) and highly sensitive T cell detection assays to confirm purity. We assess the frequency of latent HIV in monocytes using this assay in a longitudinal cohort of vsPWH (*n* = 10, 100% male, ART duration 5–14 yr) and find half of the participants showed latent HIV in monocytes. In some participants, these reservoirs could be detected over several years. Additionally, we assessed HIV genomes in monocytes from 30 vsPWH (27% male, ART duration 5–22 yr) utilizing a myeloid-adapted intact proviral DNA assay (IPDA) and demonstrate that intact genomes were present in 40% of the participants and higher total HIV DNA correlated with reactivatable latent reservoirs. The virus produced in the MDM-QVOA was capable of infecting bystander cells resulting in viral spread. These findings provide further evidence that myeloid cells meet the definition of a clinically relevant HIV reservoir and emphasize that myeloid reservoirs should be included in efforts towards an HIV cure.

## Abstract (web summary)

Detection of persistent replication-competent HIV in monocytes from virologically suppressed people with HIV indicates that monocytes have a role as a latent reservoir.

## Main

Several lines of evidence show that human immunodeficiency virus (HIV) persists in blood monocytes and tissue macrophages in virologically suppressed people with HIV (vsPWH)^1^. HIV DNA has been detected in highly purified monocytes^1–5^ and macrophages isolated from the urethra^6^, gut^7^, liver^8^ and brain^9,10^ of vsPWH. Additionally, virus from macrophage reservoirs can rebound and reseed the reservoir upon treatment interruption. Data from the Last Gift cohort show that infected brain can repopulate viral reservoirs during rebound^11^. However, little is known about the size of the myeloid (monocyte/macrophage) reservoir. Myeloid-specific restriction of HIV latency reversal and tissue localization may render the myeloid reservoir more difficult to eradicate. There are limited studies investigating whether HIV in monocytes can be reactivated to produce infectious virus in vsPWH. The few studies that have attempted to assess reactivatable reservoirs in monocytes^12,13^ often used assays not optimized for the unique biology of these cells, resulting in mixed outcomes. Currently, there are no standardized, reproducible methods to assess HIV reactivation from the monocyte reservoir and we have yet to elucidate the role monocytes play in the maintenance of tissue macrophage reservoirs. Monocytes containing replication-competent virus may reseed tissue macrophage reservoirs when they exit the blood and differentiate into monocyte-derived macrophages (MDM). Thus, we have developed an MDM quantitative viral outgrowth assay (MDM-QVOA) for HIV. We have quantitated the replication-competent and DNA MDM reservoirs in a longitudinal cohort of vsPWH and directly compared them to CD4 T cell reservoirs in the same individuals.

## Results

### Cohort characteristics

Fifteen people with HIV (PWH; 4 viremic (v) and 11 long-term virally suppressed (vs) PWH, all male) comprised the QVOA cohort. The intact proviral DNA assay (IPDA) cohort was composed of 30 vsPWH (27% male). The vsPWH used in both cohorts were on long-term suppressive ART between 5 and 22 yr and had no reported viral blips during the study period. Participants are described in Extended Data Table 1.

### MDM-QVOA development

**Fig. 1:**
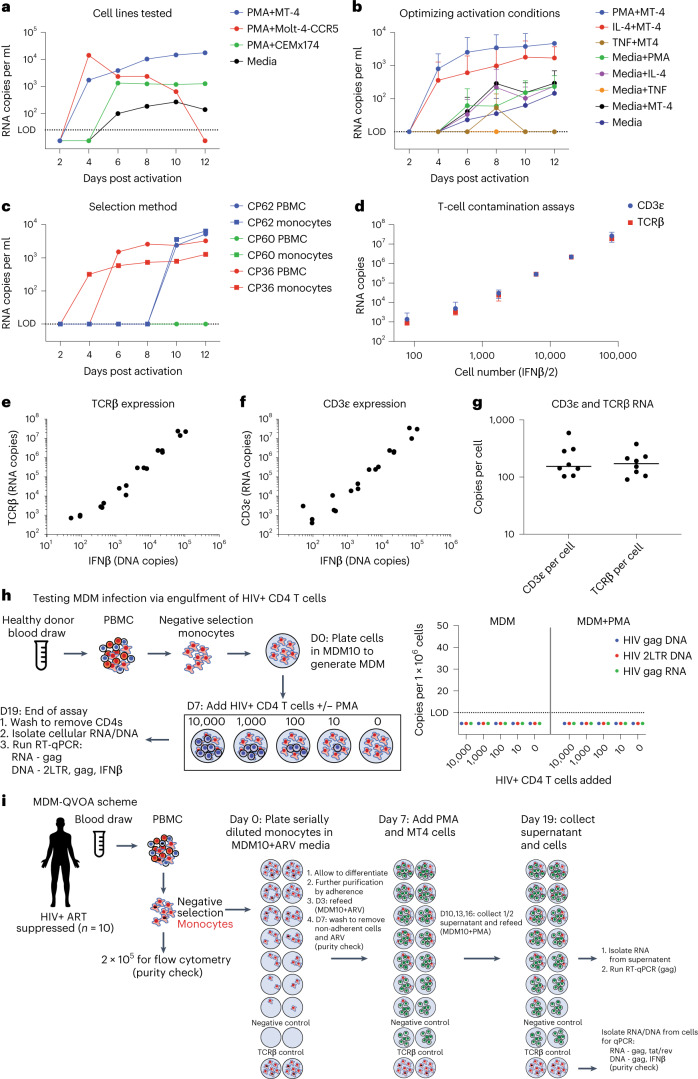
Development of the MDM-QVOA. **a**, To determine the appropriate expander cell line, one viremic vPWH was activated with PMA in the context of MT-4, Molt-4-CCR5, CEMx174 and media only, dotted line indicates limit of detection (LOD). **b**, To determine the best activation condition, 7 participants (*n* = 7, 4 vPWH and 3 virally suppressed vsPWH) were activated with PMA, IL-4, TNF𝛼 and media with and without MT-4 expander cells; mean ± s.d. **c**, To determine whether macrophages derived from negatively selected monocytes could be reactivated similarly to macrophages derived from whole PBMCs, we compared 3 participants (*n* = 3, 1 vPWH and 2 vsPWH) activated with PMA and co-cultured with MT-4. **d**–**f**, To determine the appropriate assay to detect T cell contamination, in the well or via phagocytosis, we assessed CD3ε and TCRβ RNA expression in CD4 T cells isolated from healthy donors (HD). **d**, The CD4 T cells from 3 HD were serially diluted, lysed and RNA extracted to measure TCRβ and CDε expression; mean ± s.d. TCRβ (**e**) and CDε (**f**) showed similar variability across replicates, except at the low end of the assay. **g**, CD4 T cells were isolated from 8 HD; 1 × 10^6^ CD4s per donor were lysed and assessed for CD3ε and TCRβ expression to determine the copies of each per cell; bar indicates median value. **h**, Healthy MDMs were co-cultured with HIV+ CD4 T cells from two donors (CP11 and 21) with and without PMA activation to determine whether HIV+ CD4 T cells were able to transfer viral nucleic acids to MDM; *n* = 2. **i**, A schematic of the final MDM-QVOA experimental design. Source data

**Extended Data Fig. 1:**
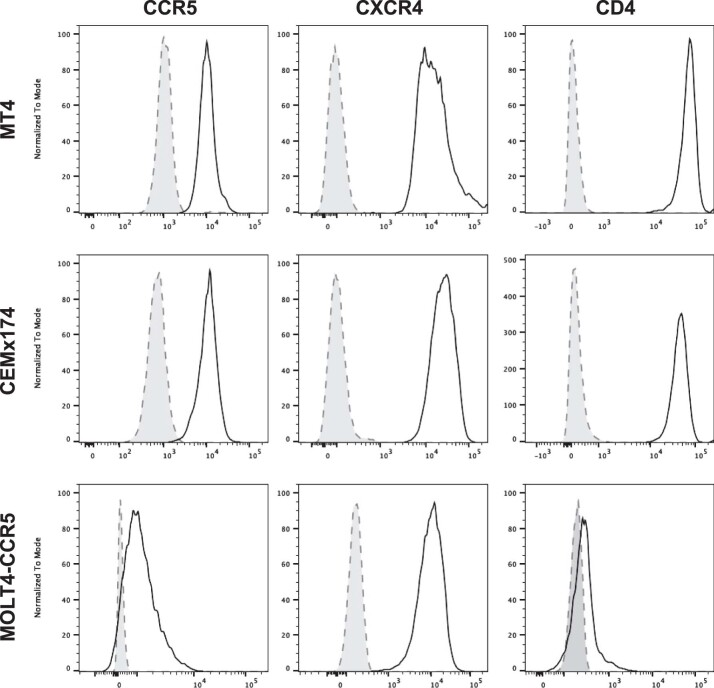
Comparison of expander cell lines for MDM-QVOA development. Cell lines, MT-4, CEMx174 and MOLT4-CCR5 were assessed for HIV entry receptor expression CCR5, CXCR4 and CD4. Cell were gated on singlets and then live cells, shaded histogram is unstained cells, black line marker of interest.

The human MDM-QVOA was developed on the basis of our previous Simian Immunodeficiency Virus (SIV)-infected ART-suppressed macaque studies^14,15^. One criticism of using the SIV model to investigate myeloid cell reservoirs is that macaques are not ART-treated for long durations compared with vsPWH. Therefore, we developed a human MDM-QVOA using blood from vsPWH. Using 1 viremic participant (CP55, Fig. 1a), we determined the appropriate expander cell line for the assay. Media alone supported viral outgrowth, but to a lesser extent. The addition of an expander cell line optimized virus propagation and detection in the assay. Monocytes were differentiated in homeostatic (M0) conditions^16^ from donor peripheral blood mononuclear cells (PBMCs) and activated using Phorbol 12-myristate 13-acetate (PMA) in the presence of MT-4, CEMx174^14,15^ and Molt-4-CCR5^17^. All cell lines had comparable baseline levels of CCR5, CXCR4 and CD4 (Extended Data Fig. 1). MT-4s promoted virus released from MDMs and supported productive infection for the duration of the assay. Molt-4-CCR5 cells began to die after 10 d in culture and CEMx174 did not propagate virus efficiently. Thus, MT-4 cells were used as the expander cell line for future MDM-QVOAs.

**Extended Data Fig. 2:**
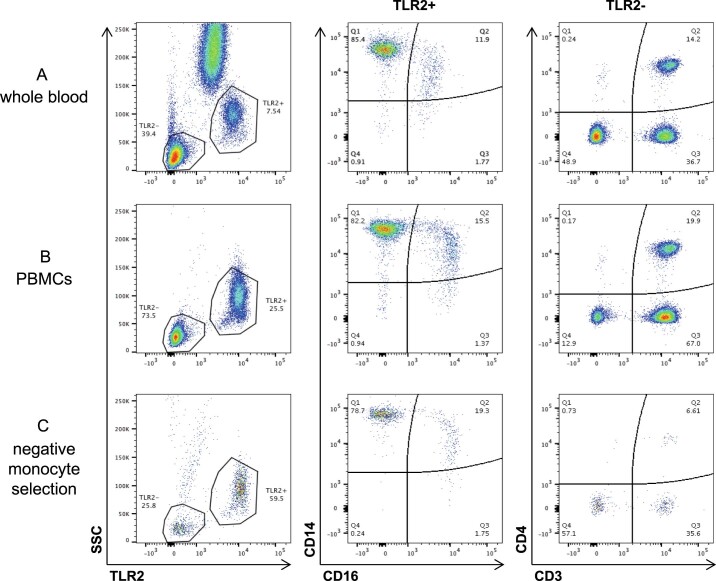
Comparison of whole blood, PBMC, negatively selected monocyte subsets. Whole blood (**A**), PBMC (**B**) and negatively selected monocytes (**C**) from one representative subject used to show that negative selection results in less CD4 contamination in cells plated and similar monocyte subset percentages. Cells are first gated on TLR2 expression, monocytes (TLR2+) are further gated by CD14 and CD16 expression, classical (CD14 + CD16−), intermediate (CD14 + CD16+) and non-classical (CD14-CD16+). Lymphocytes (TLR2-) are gate based on CD3 and CD4 expression, CD4 T cells (CD3 + CD4+).

To maximize MDM cell activation, we tested three methods of activation: PMA, TNF𝛼 and IL-4 (Fig. 1b). Using MDMs generated in M0 conditions from 7 participants (4 viremic and 3 suppressed), PMA more reliably reactivated HIV compared with TNF𝛼 and IL-4 both in the presence and absence of MT-4s. Therefore, PMA was used to activate MDMs for subsequent QVOAs. Culture conditions were established using MDMs differentiated from total PBMCs by cell adherence. However, purification of monocytes using magnetic beads was preferable to prevent CD4 uptake during the differentiation process (Extended Data Fig. 2). MDMs from purified monocytes and total PBMCs from the same donors were evaluated under the same culture conditions and were equivalent (Fig. 1c).

A primary concern of myeloid HIV assays is T cell contamination contributing to the observed signal. Therefore, we developed several checkpoints throughout the MDM-QVOA to assess the presence of T cells, as described in the methods under purity checks. Historically, we have used TCRβ RNA to determine whether T cells were present in the macaque macrophage-QVOA^14,15^. To determine whether TCRβ RNA is also appropriate for the human MDM-QVOA, we assessed both TCRβ and CD3ε^8^ RNA in purified T cells and quantitated their expression levels. We determined that TCRβ RNA measurements were more sensitive and reproducible on the low end of the assay compared with CD3ε (Fig. 1d–f). Additionally, by assessing cell number (using a single-copy gene, IFNβ) in purified CD4 T cells simultaneously with CD3ε and TCRβ RNA, we determined that we could recover a median of 150 copies of CD3ε and 174 copies of TCRβ per CD4 (CD3ε range 100–590 copies and TCRβ range 90–380 copies, Fig. 1g). Therefore, since the CD3ε assay did not markedly improve CD4 detection, we used the TCRβ RNA assay to detect T cells in subsequent QVOAs. Previous studies suggest that macrophages become infected via phagocytosis of HIV infected CD4 T cells^18–20^. To eliminate the potential of CD4 phagocytosis as the source of signal in the MDM-QVOA, we designed a control experiment to assess whether HIV+ CD4 T cells can transfer viral nucleic acids to healthy MDM in our QVOA conditions. We observed no transfer of viral nucleic acids (RNA or DNA) to MDM when co-culturing HIV+ CD4 T cells from two ART-suppressed donors (CP11 and CP21) for 12 d with and without PMA activation (Fig. 1h). This provides further evidence that minor CD4 contamination in the MDM-QVOA is not responsible for the signal observed in the assay. Evaluation of these experiments led to the development of the MDM-QVOA assay described in the methods and Fig. 1i.

### Lower levels of HIV DNA are detected in MDMs compared with CD4s

**Fig. 2:**
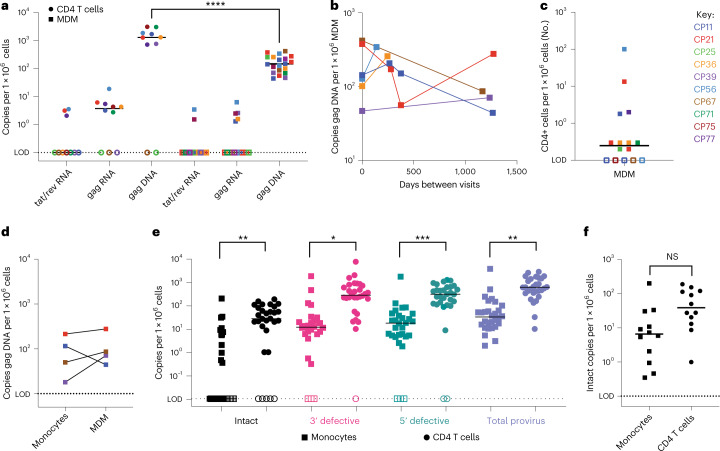
MDMs from vsPWH have consistent levels of HIV DNA over time. **a**, Ten vsPWH were assessed for HIV gag DNA, gag RNA and tat/rev RNA in isolated CD4 T cells (*n* = 10) and MDMs *(n* = 20), 6 donors were repeated 2–4 times. ****P < 0.0001, two-tailed unpaired *t*-test. **b**, HIV gag DNA in MDMs was assessed in 6 donors at multiple blood draws between 150–1,300 d apart. **c**, The number of CD4 T cells per million cells plated in MDM cultures, calculated using TCRβ RNA and CD4 percentages in whole blood; *n* = 15, 3 donors were repeated 2–3 times, line indicates median. **d**, In a subset of individuals, HIV gag DNA was assessed in monocytes and MDM from the same blood draw; *n* = 4. **e**, Monocytes and CD4 T cells were isolated from 30 vsPWH and assessed for HIV proviral DNA using IPDA. Intact, 3’ defective, 5’ defective and total proviral genome levels per million cells were compared between cell types; intact **P = 0.0014, 3’ del *P = 0.03, 5’ del ***P = 0.0005, total **P = 0.006, two-tailed unpaired *t*-test. **f**, Comparison of intact genome levels in a subset of participants that had detectable intact genomes in both CD4 and monocytes; *n* = 12, two-tailed paired *t*-test. NS, not significant. Each datapoint represents data from a specific participant, circles are CD4 data, squares are monocytes or MDM data and lines represent medians. Source data

To assess the monocyte reservoir in a small cohort of vsPWH (*n* = 10, all male), we obtained blood samples and measured HIV DNA (gag) and RNA (gag and tat/rev) in MDMs and CD4s from the same blood draw (Fig. 2a). We assessed isolated CD4s from 10 participants; all had undetectable to low levels of tat/rev (3/10 positive, median 3.1 copies per million cells), low levels of gag RNA (7/10 positive, median 4.3 copies per million cells) and high levels of gag DNA (8/10 positive, median 1,514 copies per million cells). MDMs from the same 10 participants were also assessed, and 6 of 10 participants were repeated 2–4 times for a total of 16–20 datapoints. MDMs had undetectable to low levels of tat/rev (2/16 positive, median 2 copies per million cells) and gag RNA (5/16 positive, median 2.5 copies per million cells) and low levels of gag DNA (20/20 positive, median 135.7 copies per million cells). To test variability of HIV gag DNA in MDMs over time, we assessed gag in MDM generated from 6 participants at multiple blood draws approximately 150–1,300 d apart. All DNA measures were within one log (Fig. 2b), suggesting that HIV DNA levels are stable in MDMs from these participants. On average, MDMs had 10-fold lower HIV DNA compared with their CD4 counterparts (*P* < 0.0001, Fig. 2a). All MDM samples were also assessed for T cell contamination by measuring TCRβ RNA (Fig. 2c). We observe little to no T cell contamination, with only one participant (CP56 visit 1) with 100 CD4+ T cells per million cells. This participant had a measurement of 1,885 HIV gag copies per million CD4s; thus, a contamination of 100 cells would probably contribute 0.189 copies of gag. In the MDM cell fraction, this participant had 128 copies of gag per million MDM, therefore 0.15% of the signal was contributed by CD4 T cells. Additionally, in 4 participants we assessed HIV gag DNA in monocytes before and after MDM differentiation (Fig. 2d). All (4/4) individuals had HIV gag DNA in both monocytes and MDM at similar levels. Therefore, we can confidently state that MDMs from these vsPWH contain HIV gag DNA at approximately 10-fold less than their matched CD4 T cells and at levels similar to those of monocytes before differentiation.

### Intact and defective proviral genomes are present in monocytes

To provide further evidence of the presence of an HIV DNA reservoir in monocytes, we completed IPDA^21^ on monocytes and CD4 T cells isolated at the same blood draw from 30 vsPWH (Fig. 2e). As expected, 100% of participants had detectable provirus in CD4 T cells (median 614 copies per million cells), 83% had detectable intact provirus (25/30 positive, median of detectable values 46.3 copies per million cells), 93% had detectable 5’ defective provirus (28/30 positive, median of detectable values 335 copies per million cells) and 97% had detectable 3’ defective provirus (29/30 positive, median of detectable values 295.3 copies per million cells). Additionally, 100% of participants assessed had detectable provirus in monocytes (median 32.8 copies per million cells) in at least one form, 40% had detectable intact provirus (12/30 positive, median of detectable values 6.6 copies per million cells), 90% had detectable 5’ defective (27/30 positive, median of detectable values 21 copies per million cells) and 3’ defective (27/30 positive, median of detectable values 13 copies per million cells) proviruses. The monocyte data are reported post-adjustment for CD4 T cell contamination as measured by flow cytometry at the time of isolation (described in Extended Data Table 2 and methods). Overall, CD4 T cells had higher levels of intact, 3’ defective and 5’ defective proviruses versus monocytes. However, when comparing the intact reservoir in a subset of participants that had detectable intact genomes in both cell types (12/30), the difference between cell types was no longer significant, with the median intact provirus measured at 6.6 copies per million monocytes and 38.9 copies per million CD4 T cells (*P* = 0.16, Fig. 2f). These data provide further evidence that monocytes from vsPWH contain HIV DNA genomes at lower levels compared with their CD4 T cell counterparts. This provides evidence that in a subset of vsPWH, monocytes contain intact HIV genomes that may be replication-competent upon monocyte differentiation.

### MDMs from vsPWH have persistent reactivatable reservoirs

**Fig. 3:**
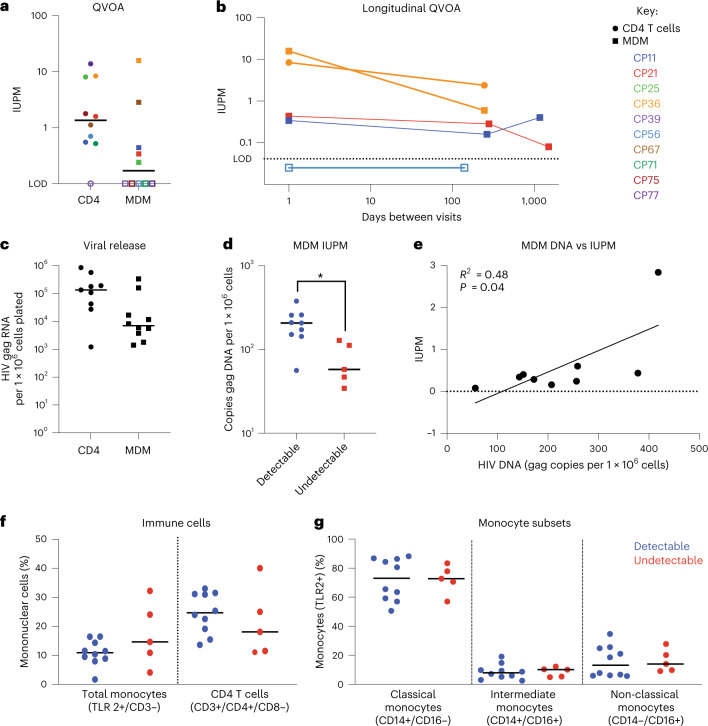
MDMs from vsPWH have reactivatable reservoirs that can be induced over time and stratify with HIV DNA burden. **a**, Ten vsPWH were assessed for reactivatable reservoirs in CD4 T cells and MDMs isolated from the same blood draw using the cell-specific CD4 and MDM QVOAs. **b**, Four participants returned for a second visit 150–280 d after the first visit. All participants had repeat MDM-QVOA completed and one participant also had a repeat CD4-QVOA completed (CP36, orange circle). Two of the 4 participants returned for a third follow-up visit 1,174 and 1,502 d after their first visit to repeat the MDM-QVOA. **c**, Average HIV gag RNA copies per million cells plated in the CD4 and MDM QVOA; *n* = 9 CD4 and *n* = 10 MDM, not significant via unpaired *t*-test. **d**, Participants with detectable IUPM values in MDM-QVOA had higher levels of HIV gag DNA compared with those with undetectable IUPM values; *n* = 9 detectable and *n* = 5 undetectable, two-tailed unpaired Student’s *t*-test *P* = 0.0122. **e**, MDM DNA levels positively correlated with MDM IUPM values; simple linear regression *R*^2^ = 0.48 and *P* = 0.04. **f**,**g**, Comparing immune cell percentages in blood from participants with detectable (*n* = 10) and undetectable (*n* = 5) IUPM values in MDM QVOA; total monocytes (TLR2+/CD3−) and CD4 T cells (**f**), and monocyte subsets (**g**). Source data

Measuring HIV DNA is not considered an accurate assessment of the replication-competent reservoir. Therefore, we assessed reactivatable reservoirs using cell-specific QVOAs (Fig. 3a). We completed CD4 T cell and MDM QVOAs on 10 vsPWH and found that 9/10 participants had reactivatable provirus in CD4 T cells (median 1.6 infectious units per million cells (IUPM)), and 5/10 had reactivatable provirus in MDM (median 0.44 IUPM). Fifty percent had inducible proviruses in the MDM cell fraction at a rate of approximately 1 in 2.5 million cells. Cell purities, input and limits of detection are shown in Extended Data Table 3.

As MDMs are differentiated from monocytes—cells that are thought to have a limited lifespan in circulation (days)^22^ and less understood lifespan in tissue (months to years)—the detection of reactivatable provirus at a single time point may not be indicative of a persistent reservoir. To determine whether MDMs contribute to the persistent HIV reservoir, meaning reproducibly reactivated over time, we obtained longitudinal blood draws from 4 participants (Fig. 3b). Three participants had detectable IUPMs (that is, reactivatable provirus) and 1 had an undetectable IUPM with the first MDM-QVOA. All (3/3) participants with reactivatable provirus at their first visit also had reactivatable provirus at their second visit. There was no significant difference in the IUPM values between visits 1 and 2 (a median of 265 d apart). Additionally, the participant with undetectable virus in the MDM-QVOA at visit 1 also had undetectable virus at visit 2 (146 d apart). As a control, we completed an additional CD4-QVOA on 1 participant and found a similar IUPM value at the second visit (Fig. 3b, CP36 orange circle). To test this further, 2 participants (CP11 and CP21) were assessed a third time using the MDM-QVOA approximately 3–4 yr after their initial visit. Both participants were on suppressive ART throughout the study with no reported viral blips and had reactivatable provirus at their third visit at similar values compared to their previous visits. To compare viral release between CD4 and MDM QVOAs, we normalized the cellular input of the assay and determined the HIV RNA copies per million cells plated. The median number of HIV RNA copies detected in all positive MDM-QVOA assays was 7.2 × 10^3^ copies per million cells (range 1.4 × 10^3^–3.5 × 10^5^ copies per million cells, Fig. 3c) compared with the CD4-QVOA assay with a median of 1.4 × 10^5^ copies per million cells (range 1.2 × 10^3^–8.9 × 10^5^ copies per million cells), and both numbers were not statistically different.

None of the MDM-QVOAs had substantial CD4 contamination as measured by flow cytometry before plating and TCRβ RNA post differentiation (Table 1 and Extended Data Table 3). Of the 16 MDM-QVOAs completed, we observed a median of 2.5% CD4 T cell contamination post selection (range 0.2–25%; Extended Data Table 3) and plated cells that were a median of 75% TLR2 positive (range 12–94.5%, TLR2 is used as a general marker of monocytes^23^; Extended Data Table 3). The remaining 25% was composed of small percentages of debris, NK and CD8 T cells that were not efficiently removed by the negative selection assay. Post differentiation, there was 1 participant with elevated levels of TCRβ RNA detected (CP56, 100 CD4 per million cells; Fig. 2e and Table 1). However, this participant did not have detectable virus in the MDM-QVOA. All other participants had a median of 0.6 (range 0–37) calculated CD4 T cells in the largest MDM-QVOA well or fewer than 13 CD4 per million cells. The percent chance of HIV+ CD4 T cells contributing to the signal observed in the MDM-QVOAs ranged from 0–0.03%. These data strongly suggest that HIV+ CD4 T cells do not contribute to the signal observed in the MDM-QVOA. Overall, these data show that MDMs not only contain reactivatable reservoirs of HIV but that these reservoirs can be reactivated over time. This supports the hypothesis that monocytes could seed tissues during viral suppression and rebound.

**Table 1:** Calculations to determine the likelihood that an HIV+ CD4 T cell contributed to assay outcome

Participant	IUPM MDM	IUPM CD4	No. CD3+ T cells in largest MDM-QVOA well	% CD3+/CD4+ cells in blood	No. CD4+ T cells in largest MDM-QVOA well	% chance of an HIV+ CD4 T cell in largest MDM-QVOA well
CP11-1	0.44	1.59	0.4^b^	53.9	0.2	0.00004
CP11-2	0.16	1.59^a^	0.7^b^	52.3	0.4	0.00006
CP11-3	0.41	1.59^a^	13.1	54.6	7.1	0.00114
CP21-1	0.34	0.55	1.1	55.9	0.6	0.00004
CP21-2	0.28	0.55^a^	0.5^b^	56	0.3	0.00002
CP21-3	0.08	0.55^a^	61.9	60	37.1	0.00206
CP25	0.24	8.08	0.8^b^	42	0.4	0.00029
CP36-1	15.8	9.50	5.2	31.5	1.7	0.00157
CP36-2	0.6	2.40	1.7	42	0.7	0.00017
CP39	<LOD	<LOD	0.4^b^	39.4	0.1	NA
CP56-1	<LOD	0.70	902.6	42.6	384.5	0.02691
CP56-2	<LOD	0.70^a^	7.8	43.3	3.4	0.00024
CP67	2.84	1.12	0.1^b^	38.4	0.0	0.00000
CP71	<LOD	0.52	3.3	40.2	1.3	0.00007
CP75	<LOD	1.78	0.2^b^	74.4	0.1	0.00002
CP77	<LOD	13.84	10.6	61.2	6.5	0.00902

^a^CD4 IUPM used from first visit for calculation.

^b^LOD was used for TCRβ calculation if results were below limit of detection.

### HIV DNA levels in MDM stratify with reactivatable reservoirs

HIV gag DNA measurements in CD4 T cells have been shown to be an overestimation of the replication-competent reservoir^24^. However, this type of analysis has never been completed for the myeloid reservoir. Therefore, we compared HIV gag DNA copies per million cells measured in the participants with IUPMs above or below the limit of detection of the MDM-QVOA. We found that participants with reactivatable provirus had higher levels of HIV DNA compared with those with undetectable virus (Fig. 3d, *P* = 0.0122). To assess whether HIV DNA levels were representative of the size of the reactivatable reservoir, we correlated HIV gag DNA copies per million cells and IUPM from MDM assays. We found that DNA and IUPM from MDMs had a weak positive correlation, with an *R*^2^ = 0.47 (Fig. 3e, *P* = 0.04). We also compared the percentages of CD4 T cells, monocytes and monocyte subsets (classical, intermediate and non-classical) in whole blood with the outcome of the MDM-QVOA. We found that there was no difference between the percentages of monocytes, CD4 T cells or monocyte subsets with reactivatable provirus vs undetectable virus in the MDM-QVOAs (Fig. 3f,g), suggesting that the presence of a particular monocyte subset does not determine the ability to reactivate the reservoir. Overall, these data demonstrate that a high total HIV DNA burden in MDMs increases the likelihood of an MDM reactivatable reservoir.

### Virus produced in MDM-QVOAs replicates in CD4 T cells

**Fig. 4:**
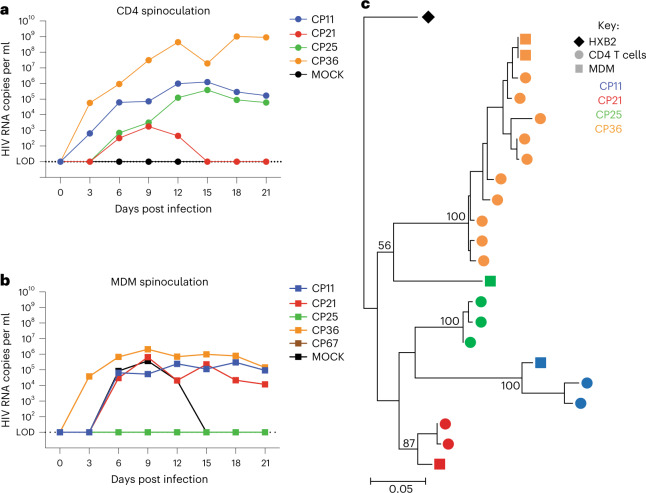
Virus released in MDM-QVOAs can spread in an activated CD4 T cell line and are distinct. **a**,**b**, QVOA culture supernatants were used to spinoculate MT-4s and determine whether viral isolates produced in the QVOAs are capable of spread. One representative positive CD4-QVOA well from 4 vsPWH (**a**) and one representative positive MDM-QVOA well from 5 vsPWH (**b**) are shown; viral input was normalized to 800 copies of HIV gag RNA per ml. **c**, The *nef* gene was sequenced at limiting dilution from positive QVOA wells in the CD4 and MDM assays. *Nef* sequences were aligned and a tree was generated using maximum likelihood estimation using the bootstrap method to test phylogeny (1,000 replications). Bootstrap outcomes are labelled at each participant node, >80 was considered significant. Each color represents a specific participant, circles indicate CD4 sequences, squares MDM sequences and diamond the reference sequence. Source data

**Extended Data Fig. 3:**
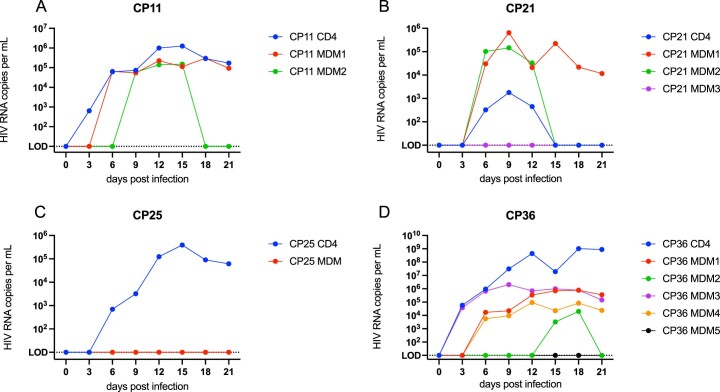
Viral replication of MDM and CD4 QVOA isolates. Culture supernatants from CP11 (**A**), CP21 (**B**), CP25 (**C**), and CP36 (**D**) positive MDM and CD4 QVOA wells were used to infect MT-4 cells and viral kinetics were assessed by measuring HIV RNA in supernatant overtime. Source data

We sought to determine whether the virus produced in the MDM-QVOA is capable of infecting CD4 T cells and therefore contribute to viral spread upon analytical treatment interruption. Using a standardized amount of HIV (800 copies gag RNA per ml) from CD4 and MDM QVOA supernatants, we spinoculated activated MT-4s and found that virus produced in MDM-QVOA is capable of infecting and expanding in a CD4 cell line similarly to viral isolates from CD4-QVOA (Fig. 4a,b). One difference observed in viral kinetics between isolates from CD4 vs MDM QVOAs was that some CD4 isolates expanded exponentially, whereas MDM isolates did not replicate exponentially in culture. Of note, there were some isolates from both QVOA assays that did not replicate (CP21 from CD4 and CP25 from MDM). We observed a variety of patterns of replication from the MDM and CD4 isolates from each participant, these patterns having been previously reported in CD4-QVOA^25^ (Extended Data Fig. 3). Overall, these data suggest that virus released from reactivated MDM reservoirs can infect bystander CD4 T cells and contribute to viral rebound post treatment interruption.

### Virus produced by MDMs is genetically distinct from CD4s

**Extended Data Fig. 4:**
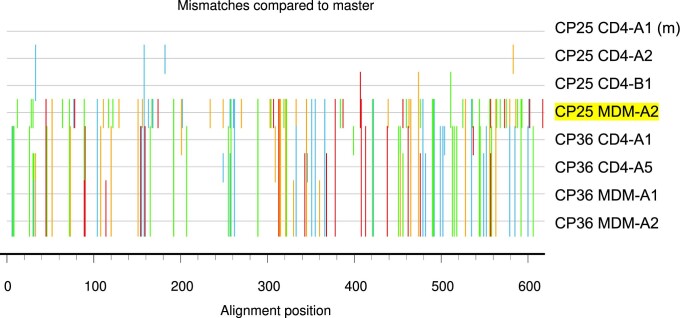
Highlighter plot comparing CP25 sequences with CP36 to rule out contamination. CP25 CD4-A1 (m) was used as the master sequence to compare the other CP25-CD4 sequences and 2 representative CP36 CD4 and MDM sequences with CP25 MDM-A2 (yellow).

**Extended Data Fig. 5:**
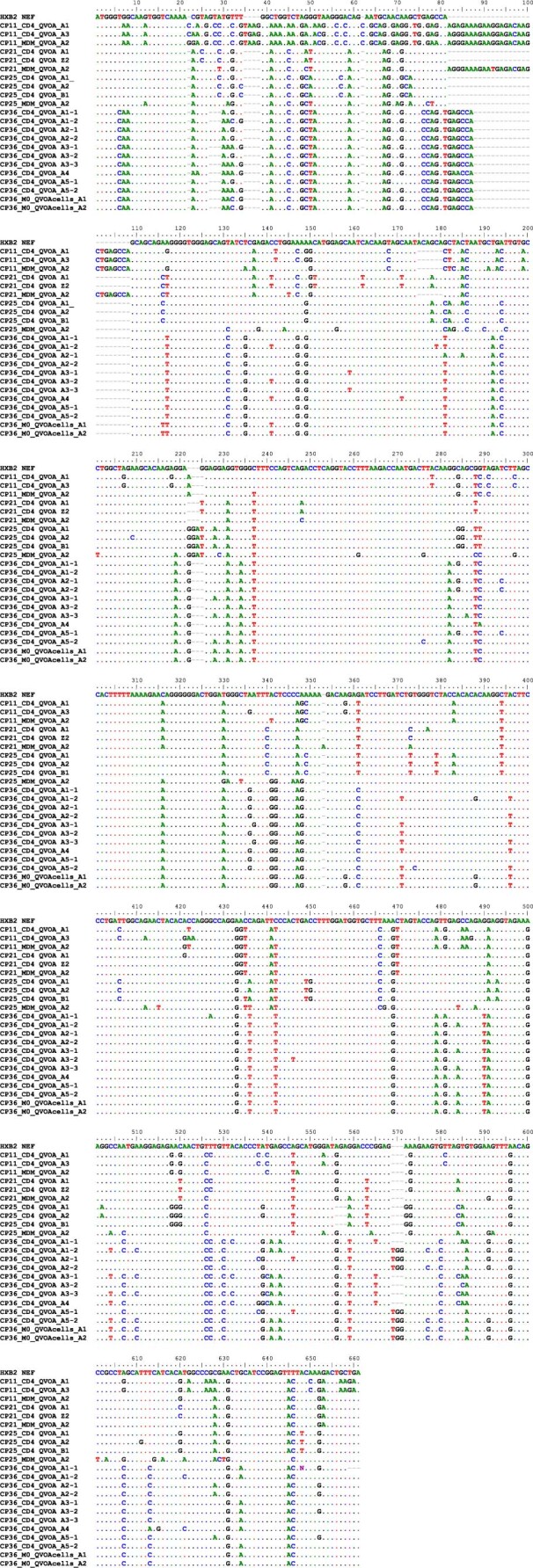
Nef sequences from positive MDM and CD4 QVOA wells.

To determine whether there are distinct HIV variants in the MDM cultures compared to the CD4 cultures, we sequenced the *nef* gene from both MDM and CD4 QVOAs from 4 participants. We found that MDM-QVOA sequences clustered with their CD4 counterparts as expected given that they were isolated from the same individual (Fig. 4c), except for CP25 whose MDM sequence clustered with a different individual (CP36) on the tree. However, the bootstrapping value was not significant (>80 considered significant), suggesting that this sequence may be randomly clustering with CP36 and is an outlier sequence. Further, when assessing the nucleotide sequence, we observed that the CP25-MDM isolate had distinct mutations from both CP25-CD4 and CP36-CD4 and MDM isolates (Extended Data Figs. 4 and 5). Therefore, we concluded that this was an accurate sequence from CP25 MDM-QVOA and not a result of contamination. This suggests that this individual might have been infected with more than one transmitted founder virus. Additionally, we sequenced *nef* from several positive MDM and CD4 QVOA wells from participant CP36. The *nef* sequences from the two positive MDM wells were identical but all CD4 *nef* sequences were distinct. Although this does not indicate clonality on the part of MDMs, as it is only a fraction of the full viral sequence, it does suggest that *nef* may be conserved in MDMs compared with CD4s as the sequences were from independent QVOA wells. These data demonstrate that reactivation of MDMs could produce distinct viruses from CD4s isolated from the same participant.

## Discussion

Persistent cellular reservoirs of latent HIV are a critical obstacle to viral eradication. Our findings demonstrate that approximately 40–50% of vsPWH harbour reactivatable latent virus in MDMs, which is notable as monocytes are disregarded in cure-based efforts. Here we quantified the monocyte reservoir in vsPWH using two independent techniques: QVOA and IPDA. The percentage of vsPWH with a monocyte reservoir is only an estimate as we may have been unable to optimally activate MDMs isolated from all participants due to differences in macrophage innate sensing^26^ and challenges in detecting HIV DNA from participants with smaller viral burdens^27–29^. Furthermore, we provide evidence that the MDM reservoir is stable and persistent in long-term vsPWH, as we measured the MDM reservoir in the same participants over a period of 9 months to 4 yr. Given that circulating monocytes have a lifespan of 72 h in blood, these data support two possible hypotheses of myeloid reservoir maintenance. First, that the bone marrow contains latent virus that seeds blood monocytes. Previous studies demonstrate that HIV can infect hematopoietic progenitor cells (bone marrow) in vivo and in vitro, causing active cytotoxic infection and latent infection^30–32^. This is supported by the detection of reactivatable virus and provirus in MDMs over time. It also suggests that we are measuring the same reservoir over time, while new monocytes are derived from the same infected progenitors. This is an understudied and divisive topic as some studies also indicate that the CD34+ cells in the bone marrow do not contain HIV provirus^33^ and purification of this cell type may be an issue^34^. The second hypothesis is that ongoing replication occurs in an unknown tissue, potentially the spleen or lymph node, leading to consistent infection of circulating monocytes. This hypothesis is supported by the finding that CD16+ monocytes are preferentially infected in vivo and ex vivo^35^, as these are the subset of monocytes thought to traverse tissues and return to circulation^36^. However, this is not supported by our data, since the monocytes require differentiation and substantial activation to produce virus in culture, and we did not detect HIV RNA in these cells, suggesting the virus is latent. Additionally, previous studies suggest a lack of ongoing viral evolution in tissues during ART, which would be expected if there was low-level replication responsible for continuous monocyte infection^37,38^. Overall, more studies are needed to determine how the monocyte reservoir is established and maintained.

Throughout the decades of HIV research, there has been evidence that myeloid cells play a role in the latent reservoir. Early ART initiation studies reported a two-phase decay in viremia in plasma, attributing the second slower phase of decay to longer-lived cells such as macrophages^39,40^. Additionally, HIV persists in monocytes and macrophages from blood and tissues as HIV DNA has been detected in highly purified monocytes^1–5^ and tissue macrophages^6–10^ from vsPWH. A recent study demonstrated novel mechanistic understanding of HIV persistence in tissue macrophages from vsPWH, suggesting that metabolic pathways may control latency in macrophages^41^. In animal models, a myeloid-only mouse model of HIV demonstrated that monocytes and macrophages can sustain infection independently of CD4 T cells^42^ and that HIV persists in tissue macrophages during ART suppression^43^. Macaque models of suppressed SIV report replication-competent myeloid reservoirs in blood and tissues from animals that have been suppressed for more than 20 months^15^. Additionally, this is not unique to HIV, as other lentiviruses preferentially infect and integrate into the host genomes of myeloid cells, creating long-lived reservoirs^44,45^. These studies highlight the importance of myeloid cells as an HIV reservoir, and further studies are needed to understand the mechanism driving their persistence and potential strategies for their elimination.

There are several limitations to this study. First, we only analysed 10 participants by MDM-QVOA and 30 participants by IPDA. Thus, our findings may not be an accurate representation of the percentage of vsPWH that contain latent HIV in monocytes. Second, we did not address latently infected tissue macrophages as these are more difficult to access in humans. Future studies that include more vsPWH, tissue macrophages and more detailed sequence analyses are needed. Despite these limitations, we provide evidence that monocytes from long-term vsPWH contain persistent latent HIV that upon reactivation is replication-competent and capable of viral spread. This study provides direct evidence that monocyte reservoirs should be included in HIV cure efforts.

## Methods

### Participants

Blood samples from healthy and HIV-positive donors were obtained with written informed consent and subsequently handled in accordance with protocols approved by the Johns Hopkins University Institutional Review Board. Cohort characteristics are reported in Extended Data Table 1.

### Flow cytometry analysis

**Extended Data Fig. 6:**
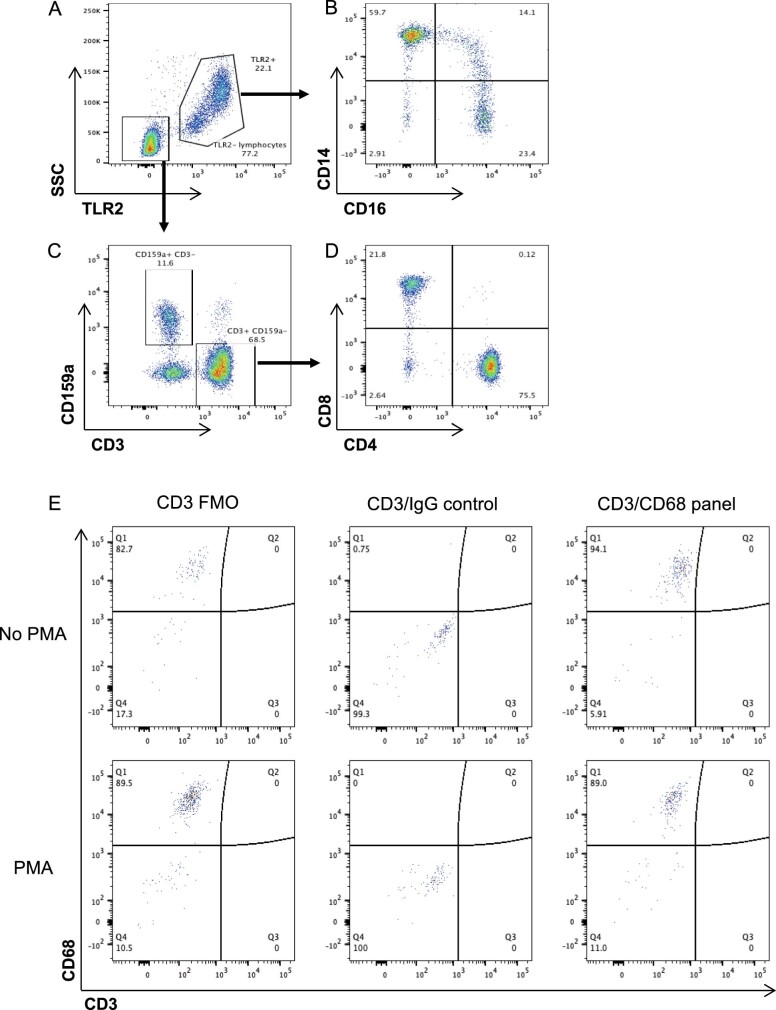
Flow cytometry gating scheme for whole blood and T cell assessment in MDM with and without activation. Post singlet gating samples are gated on TLR2 and side scatter to separate monocytes (TLR2+) from lymphocytes (TLR2−) (**A**). TLR2+ cells are then gated in monocytes subsets, classical (CD14 + CD16−), intermediate (CD14 + CD16+) and non-classical (CD14lo/-CD16+) (**B**). TLR2- cells are separated based on CD3 and CD159a expression (**C**) and then further gate on CD4 and CD8 expression (**D**). CD4 T cells are gated as (TLR2-CD3 + CD4 + CD8−). (**E**) MDM with and without activation for 12 days with PMA were removed from the plate with TrypLE and stained with Live/Dead Near IR and with or without CD3 for 30 minutes at 4C. Cells were permeabilized using Biolegend PermFast and stained with CD68 or matched IgG control. Cells were run immediately on a BD LSRFortessa. Cells were first gated to remove debris, then to remove doublets, and finally to remove dead cells.

Whole-blood samples were stained with pre-titrated antibodies using 100 μl of whole blood at room temperature for 20 min. The antibody panel and dilutions are listed in Supplementary Table 1. Whole-blood samples were then lysed and fixed in 2 ml of FACS lysing solution (BD Biosciences) for 10 min at room temperature. Samples were collected in a centrifuge at 400 × *g* for 5 min, washed in 2 ml of 1× phosphate-buffered saline (PBS) and then resuspended in 0.5 ml of PBS for analysis. Purity was assessed following selection using flow cytometry. PBMCs were stained before isolation and following pan-monocyte selection with pre-titrated monoclonal antibodies and a viability indicator. The antibody panel and dilutions are listed in Supplementary Table 1. TLR2 was used as a general monocyte cell marker as previously published^23^. PBMCs were stained, acquired and analysed as described above. Selection purities are reported in Extended Data Table 3. In select instances, purity assessments of MDM 7 d post differentiation were also completed by flow cytometry. MDMs differentiated from heathy donors were removed from the plate with TrypLE (Gibco). The antibody panel and dilutions are listed in Supplementary Table 1. In brief, cells were stained with anti-CD3 and LIVE/DEAD for 30 min at 4 °C. Cells were then permeabilized using Biolegend PermFast and stained with anti-CD68 or matched IgG control. Flow cytometry was performed on a BD LSRFortessa (BD Biosciences). Voltage settings were standardized to daily CS&T Research Bead (BD Biosciences) controls using predetermined application settings in FACSDiva 6.2 to ensure that fluorescent intensity was consistent longitudinally. Data were analysed using FlowJo 10.0.8 software (FlowJo). Representative gating strategy is shown in Extended Data Fig. 6a–d.

### Cell lines

Three lymphocyte cell lines were tested during the development of the MDM-QVOA: MT-4 cell line obtained through the NIH HIV Reagent Program, Division of AIDS (NIAID, NIH: MT-4 cells, ARP-120, contributed by Dr Douglas Richman (cat. no. 120)^46–48^); MOLT-4-CCR5 kindly donated by Dr Robert F. Siliciano from Johns Hopkins Medical School; and CEMX174 purchased from ATCC. All cell lines were propagated and maintained in R10 (RPMI 1640 medium (Gibco) supplemented with 10% heat-inactivated fetal bovine serum, 100 U of penicillin per ml and 100 µg of streptomycin per ml). The MOLT-4-CCR5 were cultured in the presence of G418 (1 mg ml^−1^) to maintain CCR5 expression. All cell lines were assessed for the necessary receptors and co-receptors for HIV entry by flow cytometry. The antibody panel and dilutions are listed in Supplementary Table 1. Antibody staining was completed as described above.

### Development of MDM-QVOA assay

Whole-blood from viremic (v) and virally suppressed people with HIV (vsPWH) was obtained for PBMCs isolation by Ficoll gradient centrifugation. The PBMCs were then used in VOAs to determine the appropriate conditions for QVOA development. All VOAs were completed on MDMs derived from fresh never-frozen PBMCs or negatively isolated monocytes (Pan Monocyte isolation kit, human; Miltenyi Biotec). PBMCs or isolated monocytes were plated at a density of 2–5 × 10^6^ cells per well and cultured in MDM10 + ARV (Dulbecco modified Eagle medium (Life Technologies) supplemented with 10% heat-inactivated human type AB serum (Gemini Bio Products)), 100 U ml^−1^ penicillin-streptomycin (Life Technologies), 20 μg ml^−1^ gentamicin (Life Technologies), 2 mM l-glutamine (Life Technologies), 2 mM sodium pyruvate (Sigma), 10 mM HEPES buffer (Life Technologies) and 50 ng ml^−1^ recombinant human macrophage colony-stimulating factor (R&D) containing anti-retroviral drugs (10 μM zidovudine (Sigma), 25 nM darunavir (Janssen) and 5 nM raltegravir (Merck)). These are considered to be M0 conditions and result in macrophage differentiation without polarization^16^. Every 3 d post plating, half of the media was removed and the cultures were replenished with fresh MDM10 + ARV. Monocytes were allowed to differentiate in these conditions for 7 d. Once MDMs were differentiated, the cells were washed twice with sterile PBS, treated with 0.025% trypsin (5 min at room temperature) and then washed twice with sterile PBS again to ensure all contaminating cells were removed and only adherent cells remained in culture. The cells were then activated with MDM10 containing one of the following activating agents: 20 ng ml^−1^ tumour necrosis factor (TNF𝛼, ProSpec), 0.5 µM ml^−1^ PMA (Sigma) and 10 ng ml^−1^ Interleukin-4 (IL-4, Prospec). Lymphocyte cell lines were added in culture to expand the virus released from infected cells. The cell lines tested in the VOAs were MT-4, MOLT-4-CCR5 and CEMX174 at a density 1 × 10^6^ per well. Assay conditions included MDM10 plus one activation reagent (PMA, TNA or IL-4) and cell line (MT-4, MOLT-4-CCR5 or CEMx174), MDM10 plus one activation reagent alone, MDM10 plus one cell line alone and MDM10 only. Supernatant (1 ml) was collected on days 2, 4, 6, 8, 10 and 12 and replaced with fresh MDM10 containing respective activation reagent or media only. Viral RNA was isolated from 1 ml of VOA supernatant at each time point using the QIAamp MinElute virus vacuum kit (Qiagen) according to the manufacturer’s recommendations, and the samples were assessed for the expression of HIV gag RNA by RT–qPCR as described below.

### MDM-QVOA assay

Reported here is the final assay used throughout the manuscript. Human PBMCs from vsPWH were isolated as described above. Two-thirds of isolated PBMCs were used for negative monocyte isolation (Pan Monocyte isolation kit, human; Miltenyi Biotec) and the remaining PBMCs reserved for CD4-QVOA assay (see CD4-QVOA assay below for details). Following pan monocyte isolation, 5 × 10^5^ cells were set aside for a purity assessment by flow cytometry, 2 × 10^6^ cells were plated for T cell control wells (1 × 10^6^ per well) and the remaining cells were plated in duplicate at 5-fold limiting dilution (Fig. 1b). All plated cells were cultured in MDM10 + ARV (described above). Monocytes were cultured for 7 d to allow for differentiation to MDMs. MDM10 + ARV was changed every 3 d to prevent viral spread in culture. On day 7, MDMs were washed 2 times with sterile PBS, 1 time with 0.025% trypsin (5 min at room temperature) and 2 more times with PBS to ensure all contaminating cells were removed and only adherent MDMs remained. MDMs were then activated with 0.5 µM ml^−1^ PMA and 1 × 10^4^–10^6^ MT-4 expander cells were added per well, excluding the T cell control wells. Supernatants were collected and replenished with newly made MDM10 + PMA every 3 d and assessed for HIV gag RNA by RT–qPCR. Supernatants from early activation time points (days 10 and 13) and supernatants from later time points (days 16 and 19) were pooled and assessed for viral RNA as described below. Cells were collected at day 19 and lysed in AllPrep buffer (RLT plus and 1% beta-mercaptoethanol, βME) for RNA and DNA isolation (see below). The frequency of cells harbouring replication-competent virus was determined using the IUPMStats v1.0 infection frequency calculator and expressed as IUPM^49^. Wells were considered positive if either the early or late time point had a cycle threshold (Ct) value less than or equal to 35 as measured by RT–qPCR. All MDM-QVOAs were assessed for CD3+ T cell contamination using RT–qPCR for TCRβ (see below).

### Purity checks to assess CD4 T cell contamination

All selected monocyte samples were analysed by flow cytometry to determine the percentage of contaminating T cells before plating. Once plated, the cells were cultured in the presence of ART for 7 d for further purification by adherence. Once the macrophages were differentiated, they were washed extensively with PBS and a low percentage of trypsin to remove any non-adherent cells. Two wells, with a minimum of 1 × 10^6^ monocytes per well, were kept as T cell controls and no MT-4s were added. At the end of the assay (day 19), the control wells were lysed and assessed for T cell contamination by qPCR for T cell receptor beta (TCRβ) RNA. The purpose of assessing TCRβ after MDM activation is to allow contaminating T cells to expand and become easier to detect. During assay development, we also assessed MDMs with and without activation for T cell contamination by flow cytometry and observed no contaminating CD3+ cells (Extended Data Fig. 6e). Additionally, CD4-QVOAs were completed on the same blood draw for all participants to act as a positive control. These measurements were then used to mathematically calculate the percent chance of HIV+ CD4 T cell contamination in the assay contributing to our positive signal (described below and Extended Data Table 4).

### Quantitation of CD3+ T cells in MDM-QVOA wells

T cell control wells without MT-4 cells were used for TCRβ RNA analyses. During assay development, we tested two methods to detect CD3+ T cells in the MDM wells: CD3ε and TCRβ. CD3ε and TCRβ RNA expression were quantified using primers, probes and reaction conditions listed in Supplementary Tables 1 and 2. All samples were quantified using target-specific RNA standard curves. In the final MDM-QVOA assay, TCRβ was used to estimate the absolute number of CD3+ T cells in MDM-QVOA (see Extended Data Table 4 for examples of how we calculated the number of CD3+ cells in the T cell control wells). In brief, we assessed the number of TCRβ RNA copies and cell number (IFNβ) in the same sample (RNA and DNA isolated via AllPrep, see below). The median number TCRβ copies per cell was determined to be 174 using CD4 T cells isolated from 10 healthy donors. Therefore, we divided the total TCRβ signal by 174 to equal CD3+ cells in the MDM well. We then used the cell number, calculated by the IFNβ signal divided by 2 (2 copies per cell), to determine the number of CD3+ cells per million. Next, we multiplied the CD3+ cells per million by the number of cells present in the largest MDM-QVOA well, as this is where we found our positive signal majority of the time. Once we had the CD3+ cells in the largest MDM-QVOA well, we multiplied this number by the percentage of CD4 T cells in whole blood at the time of draw to determine how many CD3+ cells were also CD4+. Using this number, we calculated the probability that this number of CD4 T cells could have contributed to our positive signal using the CD4 IUPM value from the same individual. The probability was then multiplied by 100 to estimate the percent chance our signal was from an HIV+ CD4 T cell.

### Control experiment to assess HIV+ CD4 transfer of viral nucleic acids

PBMCs were isolated from healthy donor whole blood and monocytes were isolated using the pan monocyte selection kit as described above. Monocytes were then plated at 500,000 cells per well and differentiated in M0 conditions for 7 d. On day 7, MDMs were washed as described above, and CD4 T cells isolated from two HIV+ donors (CP11 and 21) were added in triplicate to MDM wells (range 1 × 10^4^–10^1^cells). The MDM + HIV+ CD4 co-cultures were maintained for 12 d with and without PMA activation. On day 12, MDMs were washed and lysed, and assessed for cell-associated HIV RNA and DNA as described below.

### CD4-QVOA assay

CD4-QVOA assays were performed as previously described^17^. In brief, CD4 T cells were isolated from remaining PBMCs using a negative CD4 selection kit (Neg CD4 Kit, Miltenyi Biotec), plated at 5-fold limiting dilution and cultured in super T cell media. Cells were activated with 0.5 μg ml^−1^ phytohemagglutinin (Remel) and 10–2.5 × 10^6^ irradiated PBMCs from a heathy donor (feeders) for a minimum of 16 h. Phytohemagglutinin was then removed and 1–0.5 × 10^6^ MT-4s were added to each well. Supernatants and cells were collected on day 7. Supernatants were assessed for HIV gag RNA and cells were lysed in AllPrep buffer (RLT plus+βME) for RNA and DNA isolation (see below).

### Quantitation of HIV gag RNA in QVOA supernatants

Viral RNA was isolated from 1 ml of MDM-QVOA supernatant from each serial dilution in duplicate using the QIAamp MinElute virus vacuum kit (Qiagen) according to the manufacturer’s recommendations. Viral RNA was isolated from 0.2 ml of CD4-QVOA supernatant using the QIAamp MinElute virus spin kit (Qiagen) according to the manufacturer’s recommendations. An on-column DNase digestion was performed for all QVOA samples using the RNase-free DNase kit (Qiagen) and 3 U of RQ1 DNase (Promega), and the columns were incubated at room temperature for 20 min. Viral RNA isolated from MDM-QVOA and CD4-QVOA supernatants was assessed by RT–qPCR using the QuantiTect virus kit (Qiagen). Primers, probes and reaction conditions are listed in Supplementary Tables 2 and 3. To control for DNA contamination, one reaction was analysed without reverse transcriptase. The samples were quantified using HIV gag RNA standard curve.

### Quantification of cellular HIV gag and tat/rev RNA

HIV RNA cellular gag and tat/rev RNA genes were isolated from cells using AllPrep DNA/RNA mini kit (Qiagen) according to the manufacturer’s recommendations. Primers, probes and reaction conditions are listed in Supplementary Tables 2 and 3. The samples were quantified using target-specific RNA standard curves.

### Quantitation of HIV gag DNA

DNA samples were isolated from cells using the AllPrep DNA/RNA mini kit according to the manufacturer’s recommendations. Viral DNA was measured in the cells using the multiplex qPCR with the MP kit (Qiagen). Primers, probes and reaction conditions are listed in Supplementary Tables 2 and 3. For sample normalization and cellular quantitation, we assessed a single-copy gene, human interferon-beta (IFN-β), using primers, probes and reaction conditions listed in Supplementary Tables 1 and 2. The samples were quantified using target-specific DNA standard curves and normalized by cell number input.

### IPDA

We performed IPDA as described^21^ to separately measure genetically intact and defective (3’ deleted/hypermutated and 5’ deleted) proviral DNA, with minor modifications made for monocyte assessment. In brief, TLR2+ monocytes were isolated from participant PBMCs using the anti-biotin microbeads kit (Miltenyi Biotec) and a biotinylated TLR2 antibody (1 μg per 10^7^ cells of clone TL2.1, Invitrogen). CD4 T cells were then isolated from the remaining TLR2 negative cells using a negative CD4 selection kit (Neg CD4 kit, Miltenyi Biotec). Selected cells were then assessed for purity by flow cytometry (see above for details) and lysed in AllPrep buffer (RLT plus+βME) for DNA isolation (see above). All primers, probes and reaction conditions used for IPDA are listed in Supplementary Tables 1 and 2. Samples were run in triplicate, or if there was no signal observed, until a minimum of 1 × 10^6^ cells were acquired as determined by measuring the cellular gene RPP30. To estimate the CD4 signal that might have contributed to the results observed in the monocyte IPDA, we utilized the values assessed in the CD4 IPDA and %CD3+/CD4+ determined by flow cytometry. We mathematically calculated the number CD4 T cells present in one million monocytes, the potential intact, 3’ del or 5’ del signal in those cells and subtracted that signal from the monocyte IPDA signal. For example, sample 1 had 2% CD4 T cells in the selected monocytes and 10 intact genomes per million CD4 T cells. We would estimate that there were 20,000 CD4 T cells in 1 × 10^6^ monocytes (2 x (1 × 10^6^ cells) / 100) and 0.2 intact copies were from contaminating CD4s (10 intact / (1 × 10^6^ cells) x 20,000 CD4s), and we would then remove the latter value from the monocyte IPDA signal. The monocyte IPDA data pre and post CD4 adjustment can be found in Extended Data Table 2. All data reported in this manuscript are adjusted for CD4 but not adjusted for DNA shearing to prevent artificial increases in the intact values reported.

### In vitro infection of MT-4 with QVOA supernatants

MT-4s (2 × 10^6^) were spinoculated (2 h at 1,200 × *g*, room temperature) with 500 μl of supernatant from positive MDM or CD4 QVOA wells with available sample. Viral input was normalized to 800 copies of HIV gag for each sample. Post spinoculation, cells were washed once with sterile PBS and resuspended in 2 ml of R10, plated in a 24-well plate and incubated at 37 °C. Supernatants were collected on days 0, 3, 6, 9, 12, 15, 18 and 21 post spinoculation and fresh medium was replaced at each time point. On days 6 and 12 post spinoculation, all cultures were supplemented with an additional 1 × 10^6^ MT-4 and the spinoculation was repeated. RNA was isolated from 1 ml of sample using a QIAamp MinElute virus vacuum kit (Qiagen), and HIV *gag* RNA was quantitated by RT–qPCR as described above.

### Limiting dilution nef sequencing of QVOA virus

DNA was extracted from QVOA cells (MDM and CD4) using the AllPrep kit following the manufacturer’s recommendations. Limiting dilution PCRs to obtain clones of *nef* were performed as previously described^50,51^. In brief, DNA was used in a nested limiting dilution PCR protocol using Platinum *Taq* HiFi (Life Technologies). The outer PCRs were diluted 1:3 with deionised water, and 10 μl outer PCR DNA was used for nested amplification of full-length *nef* (661 bp). Primer sets and conditions are listed in Supplementary Tables 1 and 2 and are previously published^52^. Clonality was determined using Poisson statistics, and 2 positives per 10 wells amplified was considered clonal. PCR products were visualized using 1% agarose gels and isolated using the QIAquick gel extraction kit (Qiagen). The products were sent for Sanger sequencing. Contig sequences were generated using CodonCode aligner (v9), alignments done via Bioedit Clustal W method (v7.2) and maximum likelihood phylogenetic trees constructed using the bootstrap method to test phylogeny at 1,000 replications via MEGA software (vX). Bootstrap values greater than or equal to 80 were considered significant.

### Statistics and reproducibility

All data were analysed and graphically represented using Excel (v16.61) and/or GraphPad Prism (v9.4.1). All statistical analyses were performed using GraphPad Prism and were either unpaired *t*-tests, paired *t*-tests or one-way analyses of variance (ANOVA) with Tukey’s multiple comparisons test. Correlations were performed using simple linear regression. *P* ≤ 0.05 was considered significant. No statistical method was used to predetermine samples size, no data were excluded from analysis and data distribution was assumed to be normal, but this was not formally tested. Finally, the investigators were not blinded to allocation during experiments and outcome assessment.

### Reporting summary

Further information on research design is available in the Nature Portfolio Reporting Summary linked to this article.

### Supplementary information

Supplementary Information Supplementary Tables 1–3.

Reporting Summary

### Source data

Source Data Fig. 1 Excel file with data, with each part of the figure on a new tab.

Source Data Fig. 2 Excel file with data, with each part of the figure on a new tab.

Source Data Fig. 3 Excel file with data, with each part of the figure a new tab.

Source Data Fig. 4 Excel file with data, with each part of the figure on a new tab.

Source Data Extended Data Fig./Table 3 Excel file with data, with each part of the figure on a new tab.

## Figures and Tables

**Extended Data Table 1:**
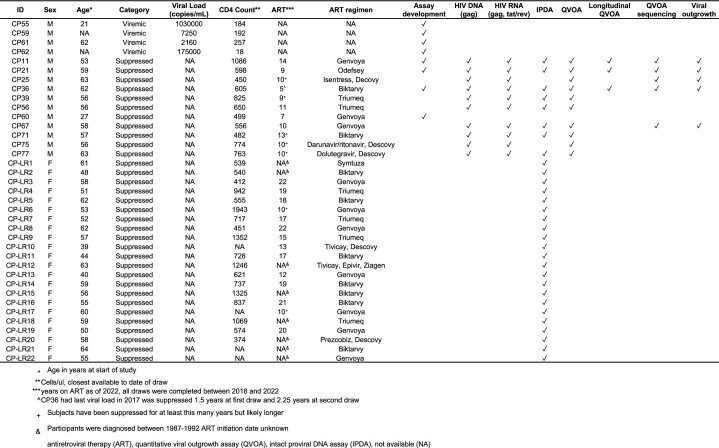
Cohort characteristics and assays throughout manuscript

**Extended Data Table 2:**
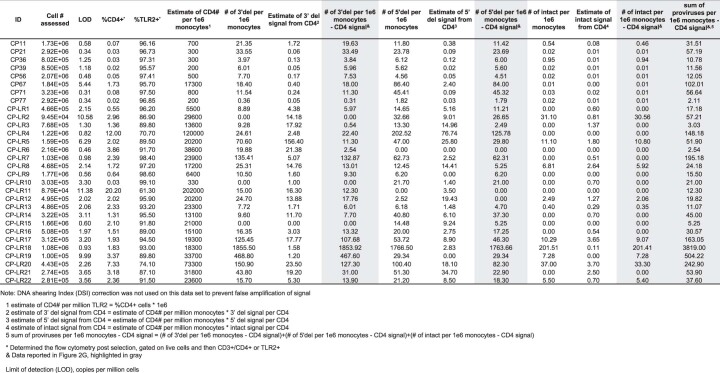
Workflow and calculations used to remove CD4 signal from monocyte IPDA analysis

**Extended Data Table 3:**
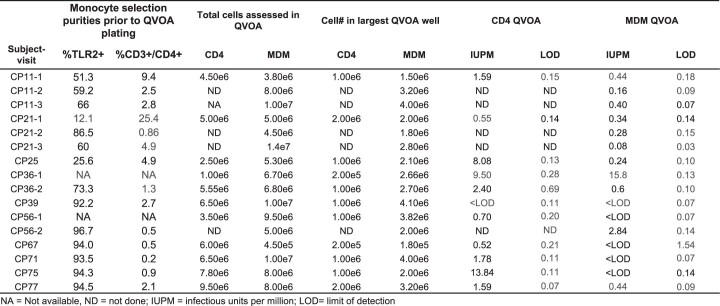
QVOA assay characteristics

**Extended Data Table 4:**
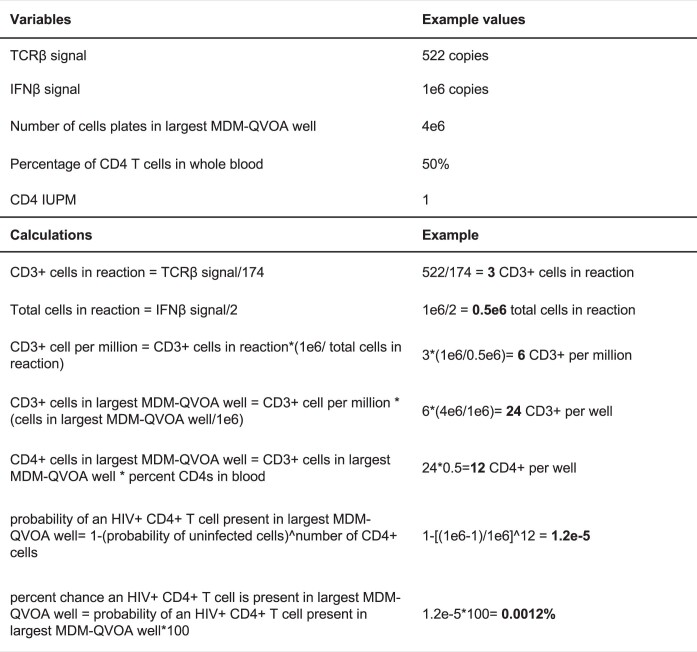
Example of CD4 contamination calculation in MDM-QVOA

## Data Availability

All sequencing data from this study have been deposited in NCBI (accession numbers OQ417114 through OQ417135). Source data are provided with this paper in Excel form.
