# Supplementary material for: Monocyte-derived macrophages contain persistent latent HIV reservoirs
Source: Nat Microbiol. Author manuscript; Available in PMC 2023 May 15. (PMC10159852; doi:10.1038/s41564-023-01349-3)
Supplement: supplemental data [file NIHMS1891209-supplement-supplemental_data.pdf]

nature microbiology

Article

https://doi.org/10.1038/s41564-023-01349-3

Monocyte-derived macrophages contain persistent latent HIV reservoirs

In the format provided by the authors and unedited

Supplementary Data

Supplemental Table 1. Flow cytometry Anitbodies

Panel	Markers	Fluorophore	Clone	Source	Cat#	dilution
Whole blood	CD3	V500	SP34-2	BD	560770	1:20
	CD4	PerCP Cy5.5	L200	BD	552838	1:05
	CD8	BV570	RPA-T8	Biolegend	301038	1:40
	CD159a	APC	Z199	Beckman Coulter	A60797	1:20
	TLR2	AF488	11G7	BD	558318	1:10
	CD14	BV650	M5E2	Biolegend	301836	1:40
	CD16	AF700	3G8	Biolegend	302026	1:40
Monocyte selection purity	CD3	V500	SP34-2	BD	560770	1:20
	CD4	PerCP Cy5.5	L200	BD	552838	1:05
	TLR2	AF488	11G7	BD	558318	1:10
	LIVE/DEAD Fixable Near-IR Dead Cell Stain Kit			Invitrogen	L10119	1:100
MDM Day 7 purity check	CD3	V500	SP34-2	BD	560770	1:20
	LIVE/DEAD Fixable Near-IR Dead Cell Stain Kit			Invitrogen	L10119	1:100
	CD68	APC	Y1/82A	Biolegend	333810	1:20
	IgG Isotype	APC	MPC-11	Biolegend	982108	1:20
Cell line receptor expression	CCR5	PE	3A9	BD	560635	1:20
	CXCR4	APC	2B11	eBioscience	17-9991-82	1:40
	CD4	BV786	L200	BD	563914	1:20

Supplemental Table 2. Primers and Probes

Assay	Primers/Probe	Source
TCRβ	F TCRβ: 5′-GAG GAC CTG AAA AAG GTG TTC-3′<br>R TCRβ: 5′-CAT AGA GGA TGG TGG CAG ACA-3′	Integrated DNA Technologies (IDT), Coralville, IA, USA
CD3ε	ProbeTCRβ: FAM 5'-ACT TCC GCT GCC AAG TCC AGT TCT AT-3′<br>F CD3ε: 5'-GTTCTCCAGAGGGTCAGATG-3'<br>R CD3ε: 5'-CAAAGGGGACAAAACAAGGAG-3'<br>Probe CD3ε: FAM 5'-AGGCCAGAATACAGGTCCCGC-3'	
HIV gag	F GAG: 5`-TCA GCC CAG AAG TAA TAC CCA TGT-3<br>R GAG: 5`-CAC TGT GTT TAG CAT GGT CTT T-3`<br>Probe GAG: FAM 5′-ATT ATC AGA AGG AGC CAC CCC ACA AGA-3′	
HIV tat/rev	F tat: 5′-ACA GTC AGA CTC ATC AAG TTT CTC TAT CAA AGC A-3′<br>R rev: 5′-GGA TCT GTC TCT GTC TCT CTC TCC ACC<br>Probe tat/rev: FAM 5′-TTC CTT CGG GCC TGT CGG GTC CC-3′	
HIV 2LTR	F 2LTR: 5'-AGA TCT GAG CCT GGG A-3'<br>R 2LTR: 5'-GTA GTT CTG CCA ATC AGG GAA G-'3<br>Probe 2LTR: HEX 5'-AGC CTC AAT AAA GCT TGC CTT GAG TGC-3'	
Human IFN-β	F IFN-β: 5`-GCC TCA AGG ACA GGA TGA ACT T-3'<br>R IFN-β: 5`- GCG TCC TCC TTC TGG AAC TG-3<br>Probe IFN-β: CY5 5`-CAT CCC TGA GGA GAT TAA GCA GCT GC-3`	
IPDA	Ψ F: 5'-CAGGACTCGGCTTGCTGAAG-3'<br>Ψ R: 5'-GCACCCATCTCTCTCCTTCTAGC-3'<br>Ψ Probe: FAM 5'-TTTTGGCGTACTCACCAGT-3' MGB<br>Env F: 5'-AGTGGTGCAGAGAGAAAAAAGAGC-3'<br>Env R: 5'-GTCTGGCCTGTACCGTCAGC-3'<br>Env intact probe: VIC 5'-CCTTGGGTTCTTGGGA-3' MGB<br>Env hypermut unlabeld probe: 5'-CCTTAGGTTCTTAGGAGC-3' MGB	IDT<br>Thermofisher<br>IDT<br>Thermofisher<br>Thermofisher
RPP30	Sequences are proprietary	Accelevir Diagnostics, Baltimore, MD
Nef Outer PCR	5'nef_out: GTAGCTGAGGGGACAGATAGGGTTAT<br>3'nef_out: GCACTCAAGGCAAGCTTTATTGAGGC	IDT
Nef Inner PCR (and sequencing)	5nef_inn: CGTCTAGAACATACCTAGAAGAATAAGACAGG<br>3nef_inn: CGGAATCCGTCCCCAGCGGAAAGTCCCTTGTA	

Supplemental Table 3. Cycling conditions

Assay	Temp	time	cycles	Instrument
TCRβ, CD3ε RNA	50°C	30 min	1x	CFX96 Real-Time PCR Detection System (Bio-Rad)
	95°C	15 min	1x	
	94°C	15s	45x	
	55°C	15s		
	60°C	30s		
HIV tat/rev RNA	50°C	30 min	1x	
	95°C	5 min	1x	
	95°C	15s	45x	
	55°C	15s		
	60°C	30s		
HIV gag, 2LTR, IFNβ multiplex DNA	95°C	10 min	1x	
	95°C	15s	45x	
	55°C	15s		
	60°C	30s		
IPDA/RPP30	95°C	10 min	1x	QX200 Droplet Digital System (Biorad)
	94°C	30s	45x	
	60°C	1 min		
	98°C	10 min	1x	
	12°C	Infinite	1x	
	*all steps ramp rate 2C/s			
Nef Outer and Inner Limiting dilution PCRs	94°C	3 min	1x	Biorad T100 Thermocycler
	94°C	30s	45x	
	55°C	30s		
	68°C	1 min		
	68°C	5 min	1x	
	4°C	Hold		
