# Supplementary material for: Monocyte-derived macrophages contain persistent latent HIV reservoirs
Source: Nat Microbiol. 2023 Mar 27;8(5):833–44. doi: 10.1038/s41564-023-01349-3 (PMC10159852; doi:10.1038/s41564-023-01349-3)
Supplement: Supplementary file 1 [file 41564_2023_1349_MOESM2_ESM.pdf]

nature portfolio

Corresponding author(s): Janice Clements

Last updated by author(s): 2/08/2023

Reporting Summary

Nature Portfolio wishes to improve the reproducibility of the work that we publish. This form provides structure for consistency and transparency in reporting. For further information on Nature Portfolio policies, see our Editorial Policies and the Editorial Policy Checklist.

Statistics

For all statistical analyses, confirm that the following items are present in the figure legend, table legend, main text, or Methods section.

n/a	Confirmed	
☐	☒	The exact sample size (n) for each experimental group/condition, given as a discrete number and unit of measurement
☐	☒	A statement on whether measurements were taken from distinct samples or whether the same sample was measured repeatedly
☐	☒	The statistical test(s) used AND whether they are one- or two-sided<br>*Only common tests should be described solely by name; describe more complex techniques in the Methods section.*
☒	☐	A description of all covariates tested
☒	☐	A description of any assumptions or corrections, such as tests of normality and adjustment for multiple comparisons
☐	☒	A full description of the statistical parameters including central tendency (e.g. means) or other basic estimates (e.g. regression coefficient) AND variation (e.g. standard deviation) or associated estimates of uncertainty (e.g. confidence intervals)
☐	☒	For null hypothesis testing, the test statistic (e.g. F, t, r) with confidence intervals, effect sizes, degrees of freedom and P value noted<br>*Give P values as exact values whenever suitable.*
☒	☐	For Bayesian analysis, information on the choice of priors and Markov chain Monte Carlo settings
☒	☐	For hierarchical and complex designs, identification of the appropriate level for tests and full reporting of outcomes
☒	☐	Estimates of effect sizes (e.g. Cohen's d, Pearson's r), indicating how they were calculated

Our web collection on statistics for biologists contains articles on many of the points above.

Software and code

Policy information about availability of computer code

Data collection	N/A
Data analysis	Excel (version 16.61), Graphpad prism (version 9.3.1 (350), Flow Jo (10.0.8), Codon Code (version 9.0.1), Bioedit (version 7.2), Mega (version X) programs were used for all analysis or source data in this manuscript

For manuscripts utilizing custom algorithms or software that are central to the research but not yet described in published literature, software must be made available to editors and reviewers. We strongly encourage code deposition in a community repository (e.g. GitHub). See the Nature Portfolio guidelines for submitting code & software for further information.

Data

Policy information about availability of data

All manuscripts must include a data availability statement. This statement should provide the following information, where applicable:

- Accession codes, unique identifiers, or web links for publicly available datasets
- A description of any restrictions on data availability
- For clinical datasets or third party data, please ensure that the statement adheres to our policy

All sequencing data from this study have been deposited in NCBI (OQ417114-OQ417135). Source data are provided with this paper in Excel form.

Human research participants

Policy information about studies involving human research participants and Sex and Gender in Research.

Reporting on sex and gender	This study includes: Quantitative Viral Outgrowth analyses of male participants and Intact Proviral DNA Analyses of male and female participants . Descriptions of both assays are described in supplemental data. All participants had HIV diagnosis and were on long-term suppressive ART. The reason for limiting the QVOA study to males was that innate immune cell signaling and function varies significantly between males and females. The goal was to limit variability as much as possible while still utilizing primary human samples. The IPDA was performed with both male and female participants as innate sensing is not an issue.
Population characteristics	Subjects were males and females, required an HIV diagnosis on long-term suppressive ART (4-22 years), median age was 57 years (range 21-64) , no genetic characteristics were obtain as a part of the study, ART regimen was reported.
Recruitment	The participants were recruited from two cohorts of study participants who were involved in ongoing latent reservoir studies. All subjects were chosen based on interest in the study and if they were on long-term suppressive ART regimens. A certain age range was not a requirement, but participants younger that 18 years of age were not included in the original cohorts. The participants received monetary compensation but this is unlikely to have impacted our results.
Ethics oversight	Johns Hopkins University Institutional Review Board

Note that full information on the approval of the study protocol must also be provided in the manuscript.

Field-specific reporting

Please select the one below that is the best fit for your research. If you are not sure, read the appropriate sections before making your selection.

☒ Life sciences ☐ Behavioural & social sciences ☐ Ecological, evolutionary & environmental sciences

For a reference copy of the document with all sections, see nature.com/documents/nr-reporting-summary-flat.pdf

Life sciences study design

All studies must disclose on these points even when the disclosure is negative.

Sample size	Whole blood was obtained from de-identified participants with HIV and on longterm suppressive ART. 30 subjects were used for cross-sectional IPDA analysis, 10 subjects were used for cross-sectional QVOA analysis, and 4 subjects for longitudinal analysis. Initial sample sizes for crossectional analysis were determined based on our ability to recruit individuals from our hospital clinic. No statistical calculation was used. Samples size for longitudinal observation were determined based results from the cross sectional study. Despite having smaller sample sizes we were able to observe significant differences in IPDA and QVOA analyses. Future studies should include larger populations.
Data exclusions	None
Replication	Steps to develop the assay were completed with various #s of repeats. Expander cell line, only one experiment with one donor; Activation: completed on 7 distinct donors; Selection method: 3 distinct donors, T cell contamination assays 8 healthy donors. All HIV DNA and RNA assays on MDM or CD4s were setup with 3 technical replicates, select participants that were available for longitudinal follow up had 2-4 additional Total HIV DNA assays completed on MDMs 6 months to 4 years later to determine stability. IPDA on monocytes and CD4s were performed with 3-6 replicates to ensure a minimum of 1 million cells from each cell type was assessed per participant. All cross-sectional QVOAs were setup in duplicate (technical replicates). Select participants that were available for longitudinal followup had 2-3 additional MDM-QVOAs completed 6 months to 4 years later to confirm that the positive signal observed in the cross-sectional experiment were stable and present longterm. Viral kintetic assays were setup in duplicate (technical replicates) for all positive MDM-QVOA and corresponding CD4-QVOA. Sequencing was completed at limiting dilution with the attempt to get as many distinct sequences as possible, however, often there was only one.
Randomization	This is not applicable, all samples were treated exactly the same. The goal of this study was to determine if long-term suppressed contian myeloid reservoirs.
Blinding	All samples were de-identified clinical samples given to us from our clinical collaborator and considered to be one de-identified group. Therefore group blinding was not necessary becasue we had no way of knowing which participants would have active myeloid reservoirs. For viral kinetic experiments, which involve viral supernatant from QVOA we were not blinded to the QVOA it was collected from because blinding is not needed for this type of experiment. For sequencing experiments, we were not blinded because once again blinding is not relevant to the outcome.

Reporting for specific materials, systems and methods

We require information from authors about some types of materials, experimental systems and methods used in many studies. Here, indicate whether each material, system or method listed is relevant to your study. If you are not sure if a list item applies to your research, read the appropriate section before selecting a response.

Materials & experimental systems

n/a	Involved in the study
☐	☒ Antibodies
☐	☒ Eukaryotic cell lines
☒	☐ Palaeontology and archaeology
☒	☐ Animals and other organisms
☒	☐ Clinical data
☒	☐ Dual use research of concern

Methods

n/a	Involved in the study
☒	☐ ChIP-seq
☐	☒ Flow cytometry
☒	☐ MRI-based neuroimaging

Antibodies

Antibodies used	CD3 V500 SP34-2 BD #560770, CD4 PerCP Cy5.5 L200 BD #552838, CD8 BV570 RPA-T8 Biolegend #301038, CD159a APC Z199 Beckman Coulter #A60797, TLR2 AF488 11G7 #BD 558318, CD14 BV650 M5E2 Biolegend #301836, CD16 AF700 3G8 Biolegend #302026, LIVE/DEAD Fixable Near-IR Dead Cell Stain Kit Invitrogen #L10119, CD68 APC Y1/82A Biolegend #333810, IgG Isotype APC MPC-11 Biolegend #982108, CCR5 PE 3A9 BD #560635, CXCR4 APC 2B11 eBioscience #17-9991-82, CD4 BV786 L200 BD #563914
Validation	All antibodies were purchased based on manufacture statements that indicated the were reactive to human samples. Once received they antibodies were titrated on whole blood and tested along side fluorescence minus one (FMO) and unstained controls to confirm specificity. Dilutions were determine based on the lowest titration that maintained signal and are listed in supplemental table 1.

Eukaryotic cell lines

Policy information about cell lines and Sex and Gender in Research

Cell line source(s)	Three lymphocyte cell-lines were tested during the development of the MDM-QVOA: MT-4 cell-line obtained through the NIH HIV Reagent Program, Division of AIDS, NIAID, NIH: MT-4 Cells, ARP-120, contributed by Dr. Douglas Richman (cat#120). MOLT-4-CCR5 were kindly donated by Dr. Robert. F Siliciano from Johns Hopkins Medical School. The CEMX174 were purchased from ATCC, US.
Authentication	Cell lines were stained for the necessary cell receptors needed for viral entry prior to using in the study
Mycoplasma contamination	Not tested for mycoplasma contamination directly, however the lab undergoes mycoplasma testing yearly.
Commonly misidentified lines (See ICLAC register)	None

Flow Cytometry

Plots

Confirm that:

☒ The axis labels state the marker and fluorochrome used (e.g. CD4-FITC).

☒ The axis scales are clearly visible. Include numbers along axes only for bottom left plot of group (a 'group' is an analysis of identical markers).

☒ All plots are contour plots with outliers or pseudocolor plots.

☒ A numerical value for number of cells or percentage (with statistics) is provided.

Methodology

Sample preparation	Whole-blood samples were stained with pretitrated amounts of monoclonal antibodies using 100µl of whole blood at room temperature for 20 min. Whole-blood samples were then lysed and fixed in 2 mL of FACS Lysing Solution (BD Biosciences, San Jose, CA) for 10 min at room temperature. Samples were collected in a centrifuge at 400×g for 5 min, washed in 2 mL of 1× phosphate-buffered saline (PBS), and then resuspended in 0.5 mL of PBS for analysis.
Instrument	Flow cytometry was performed on a BD LSRFortessa (BD Biosciences, San Jose, CA). Voltage settings were standardized to daily CS&T Research Beads (BD Biosciences, San Jose, CA) controls using predetermined application settings in FACSDiva 6.2 to ensure fluorescent intensity was consistent longitudinally.
Software	Data were analyzed using FlowJo 10.0.8 software (FlowJo, LLC, Ashland, OR).
Cell population abundance	Samples were not sorted only assessed for phenotype via flow cytometry.

Gating strategy

Post singlet gating (SSC-W vs SSC-H) samples were gated on TLR2 and side scatter (SSC) to separate monocytes (TLR2+) from lymphocytes (TLR2-). TLR2+ cells are then gated in monocytes subsets, classical (CD14+CD16-), intermediate (CD14+CD16+) and non-classical (CD14lo/-CD16+). TLR2- cells are separated based on CD3 and CD159a expressionand then further gate on CD4 and CD8 expression. CD4 T cells are gated as (TLR2-CD3+CD4+CD8-)

☒ Tick this box to confirm that a figure exemplifying the gating strategy is provided in the Supplementary Information.
